# The Integrated HIV-1 Provirus in Patient Sperm Chromosome and Its Transfer into the Early Embryo by Fertilization

**DOI:** 10.1371/journal.pone.0028586

**Published:** 2011-12-14

**Authors:** Dian Wang, Lian-Bing Li, Zhi-Wei Hou, Xiang-Jin Kang, Qing-Dong Xie, Xiao-jun Yu, Ming-Fu Ma, Bo-Lu Ma, Zheng-Song Wang, Yong Lei, Tian-Hua Huang

**Affiliations:** 1 Research Center for Reproductive Medicine, Shantou University Medical College, Shantou, China; 2 Chongqing Key Laboratory of Birth Defects and Reproductive Health, Chongqing, China; 3 Jiangbei District Center for Disease Control and Prevention, Chongqing, China; 4 Forensic Medicine Department, Shantou University Medical College, Shantou, China; University of California San Francisco, United States of America

## Abstract

Complete understanding of the route of HIV-1 transmission is an important prerequisite for curbing the HIV/AIDS pandemic. So far, the known routes of HIV-1 transmission include sexual contact, needle sharing, puncture, transfusion and mother-to-child transmission. Whether HIV can be vertically transmitted from human sperm to embryo by fertilization is largely undetermined. Direct research on embryo derived from infected human sperm and healthy human ova have been difficult because of ethical issues and problems in the collection of ova. However, the use of inter-specific *in vitro fertilization* (IVF) between human sperm and hamster ova can avoid both of these problems. Combined with molecular, cytogenetical and immunological techniques such as the preparation of human sperm chromosomes, fluorescent in situ hybridization (FISH), and immunofluorescence assay (IFA), this study mainly explored whether any integrated HIV provirus were present in the chromosomes of infected patients' sperm, and whether that provirus could be transferred into early embryos by fertilization and maintain its function of replication and expression. Evidence showed that HIV-1 nucleic acid was present in the spermatozoa of HIV/AIDS patients, that HIV-1 provirus is present on the patient sperm chromosome, that the integrated provirus could be transferred into early embryo chromosomally integrated by fertilization, and that it could replicate alongside the embryonic genome and subsequently express its protein in the embryo. These findings indicate the possibility of vertical transmission of HIV-1 from the sperm genome to the embryonic genome by fertilization. This study also offers a platform for the research into this new mode of transmission for other viruses, especially sexually transmitted viruses.

## Introduction

Sexual transmission remains the major mode of HIV transmission, and semen is the main vehicle. This is because it contains free and cell-associated virions [1]–[3]. Whether human spermatozoa, the main ingredient of semen, participate in HIV transmission is a crucial topic because the spermatozoa are the germ cells that fertilize ova and transfer male genetic information to progeny [3]. This prompts us to go back to the primary question of whether the spermatozoa in male HIV/AIDS patients carry HIV-1. Despite the initial long debate, the presence of viral particles and nucleic acids in spermatozoa from HIV-1-infected men was finally confirmed using a variety of techniques [4]–[9]. Horizontal transmission and vertical transmission of HIV by spermatozoa have been demonstrated by two separate research groups, and both indicated that the virus is transmitted through cell-to-cell contact [3], [10]. It is still unclear whether HIV-positive spermatozoa and embryos are capable of producing infectious viral particle, capable of completing of the HIV life cycle. Direct examination of the HIV life cycle in human spermatozoa and embryos derived from these spermatozoa is not easy because there are several processes that are difficult to detect. These include proviral integration, viral replication, and the fertilization process [11], [12]. However, this complex problem can be broken down by examining various steps of HIV lifecycle independently. In this project, several key lifecycle processes related to the vertical transmission of HIV in human spermatozoa were investigated separately: the integration of HIV DNA into the genome of sperm, the transfer of sperm HIV DNA into the early embryo, and the replication and expression of sperm-introduced HIV genes in early embryo. Semen was collected from HIV/AIDS patients and subjected to inter-specific IVF and a combination of molecular, cytogenetical and immunological techniques, including the preparation of human sperm chromosomes and 2-cell nuclei, fluorescence in situ hybridization (FISH), and immunofluorescence assay (IFA). We are able to address the essential problems described above regarding the vertical transmissibility of HIV in spermatozoa from HIV/AIDS patients.

## Results

### Presence of HIV-1 in the spermatozoa of HIV/AIDS patients

**Figure 1:**
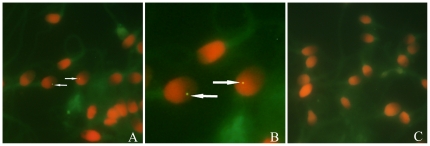
Detection of HIV-1 in spermatozoa by FISH with biotinylated HIV gag and HIV pol probes. Spermatozoa from HIV/AIDS patients and healthy donors are subjected to FISH to determine the presence of HIV nucleic acids. Propidine iodide (PI) was used to stain the nuclei of spermatozoa (salmon pink). A: Positive FISH signals on the head of sperm from an HIV/AIDS patient (arrow); B: Magnification of the partial A; C: Absence of positive signal on sperm from a healthy donor. Magnification:×1000 (A, C).

To confirm the presence of HIV-1 in the spermatozoa of HIV/AIDS patients, FISH was performed on spermatozoa from the patients and from the healthy donors. Spermatozoa were incubated with both probes for HIV-1 gag and HIV-1 pol DNA and then washed and incubated with FITC anti-biotin. Positive FITC signals were detected in the heads of spermatozoa from 9 (27.2%) of 33 HIV/AIDS patients (Fig. 1-a), and 33 of 2745 spermatozoa from 9 FISH-positive patients showed the positive signals for HIV-1 gag and HIV-1 pol DNA. The percentage of spermatozoa with positive FISH signals in the group of FISH-positive patients was 1.33%. The reliability of HIV-1 DNA detection by FISH was confirmed by the specificity and sensitivity controls and by using two different probes represents the viral genes of gag and pol. No positive signals for HIV-1 gag and pol DNA was observed in sperm slides from three HIV-seronegative men.

### Presence of HIV-1 provirus on sperm chromosome of HIV/AIDS patients

**Figure 2:**
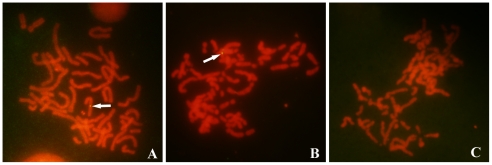
Detection of HIV-1 provirus on chromosomes by FISH. Chromosomes of spermatozoa from HIV/AIDS patients and healthy donors were prepared. FISH was performed to determine the HIV provirus sequence in these chromosomes. A: HIV provirus (arrow) on a peripheral blood mononuclear cell (PBMC) chromosome from an AIDS patient (arrow, positive control); B: HIV provirus (arrow) on sperm chromosomes from an AIDS patient; C: Absence of provirus on sperm chromosomes from a healthy male (negative control). Magnification: ×1000 (A, B, C).

To detect the integration of HIV-1 provirus in sperm chromosome of HIV/AIDS patients, we first prepared the metaphase chromosomes of spermatozoa and peripheral blood mononuclear cells (PBMC) from HIV^+^ patients and healthy donors. One hundred and fifteen sperm chromosome complements were successfully obtained. Ninety of them were subjected to FISH to detect the integrated HIV provirus on them. Positive FISH signals for HIV-1 gag and pol DNA were observed in two sperm chromosome complements (2.2%) from patients (Fig. 2A), indicating the presence of the integration of HIV provirus into sperm metaphase chromosomes. No signal was detected in spermatozoa from any of healthy donors (Fig. 2C). FISH on 172 (PBMC) metaphase chromosomes from three infected men revealed 3.48% (6 out of 172) to be positive for HIV provirus (Fig. 2B).

### Transfer of HIV-1 into ova and its replication in early embryos

To determine the presence and replication pattern of HIV-1 in early embryonic cells, we first prepared the nuclei of 2-cell embryos derived from zona-free hamster ova *in vitro* fertilized with spermatozoa from HIV/AIDS patients. The overall fertilization rate ranged from 0% to 61.3% due to the ability of spermatozoa from different patients to complete fertilization. The nuclei of 56 two-cell embryos were analyzed using FISH.

**Figure 3:**
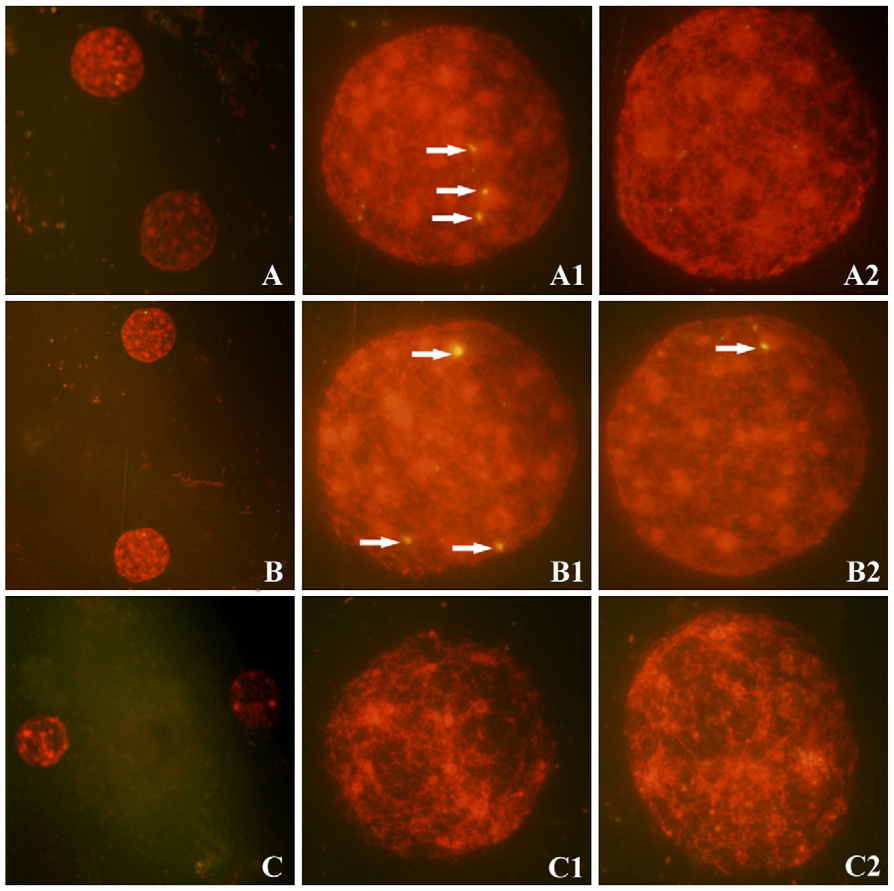
Detection of HIV-1 sequences in nuclei of two-cell embryos by FISH. A: Two nuclei of a 2-cell embryo derived from the sperm of an AIDS patient. A1 and A2 are magnifications of the nuclei in A. HIV-1 provirus was found in one nucleus of a two-cell embryo (A1, arrows). B: two nuclei of a 2-cell embryo derived from the sperm of an AIDS patient. B1 and B2 are magnifications of the nuclei in B. HIV-1 provirus was identified in each nucleus (arrows). C: No provirus was detected in the nuclei of 2-cell embryos derived from the sperm of a healthy donor. C1 and C2 are magnifications of the nuclei in C. Magnification: A, B, C (×200); A1, A2, B1, B2, C1, C2 (×1000).

Positive signals for HIV-1 gag and pol DNA were detected in one of two nuclei from three 2-cell embryos (Fig. 3A) and in both two nuclei from one 2-cell embryo (Fig. 3A). These results indicated the presence of integrated HIV-1 provirus in the nuclei of two-cell embryos and its replication along with replication of the host genome. No positive signals were detected in the nuclei of embryos derived from zona-free hamster ova *in vitro* fertilized with spermatozoa from healthy donors (Fig. 3C).

### Expression of HIV-1 P24 in patient spermatozoa and 2-cell embryos

**Figure 4:**
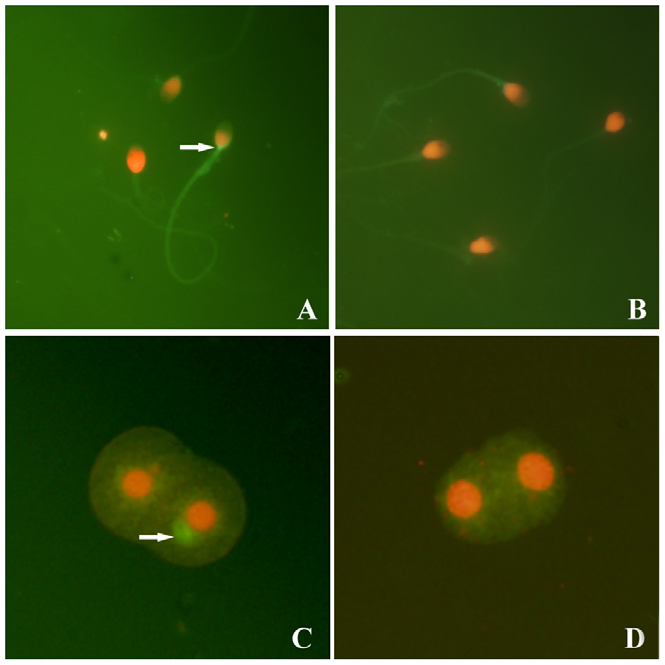
Immunofluorescence of HIV p24 in sperm and 2-cell embryos. A: p24 in sperm from an AIDS patient (arrow); B: No p24 was detected in sperm from a healthy donor; C: p24 within the cytoplasm of an embryonic cell derived from sperm of an HIV/AIDS patient (arrow); D: No p24 was detected in an embryonic cell derived from the sperm of a healthy donor. Magnification: A, B, (×200); C,D (×1000).

To determine the presence of HIV-1 gene expression in spermatozoa and 2-cell embryos, IFAs were performed on inter-phase spermatozoa and the 2-cell embryos. Positive signals for HIV-1 p24 were observed in spermatozoa from 3 of the 33 HIV/AIDS patients. Ninety one 2-cell embryos derived from zona-free hamster ova *in vitro* fertilized with spermatozoa of HIV/AIDS patients were collected and examined by FISH. Of these, 2 showed signals for HIV-1 p24.(2.2%, Fig. 4C). No positive signals were observed in the sperm from healthy donors (Fig. 4B) or in the embryos derived from spermatozoa from healthy donors (Fig. 4D).

## Discussion

Previous studies on the detection of HIV-1 in spermatozoa from HIV/AIDS patients showed that the more sensitive the method used, the greater the possibility of detecting HIV [4], [8]. The main reason for the negative results of the previous studies may be due to the lower sensitivity of the methods used for the viral detection. We used a highly sensitive method, FISH, to detect HIV-1 nucleic acid in patient spermatozoa. Two sequences representing HIV-1 gag and HIV-1 pol, which contain nearly half of the HIV-1 genome, were used as double probes. As an in situ detection method, FISH can easily exclude the false positives from seminal plasma and non-seminal cells (NSC) which may have been a considerable problem with PCR method. Positive FISH signals were observed in the spermatozoa of 9 patients (27.2%), which confirmed the presence of HIV-1 in patient spermatozoa. Though the classical receptor of HIV CD4 has not been detected in human mature spermatozoa, alternative receptor GalAAG, a glycolipid related to galactosylceramide, and CCR5 have been found in ejaculated spermatozoa [13]–[16]. Mammalian spermatozoa are capable of spontaneously taking up foreign DNA and RNA, including viral nucleic acids spontaneously [17], [18]. These findings provide a solid basis for the current idea that human spermatozoa can carry HIV-1 nucleic acids. Both events occurred in the absence of seminal plasma [4]. This means that the male germ cells can take up HIV-1 nucleic acids or are infected by the virus during their time in epididymis or testes.

### The integration of HIV-1 provirus into the genome of patient sperm

For HIV, and all other retrovirus, integration of HIV into the host genome is a prerequisite for efficient replication of virus in host cells. It is a key part of the HIV life cycle [19]. Direct investigation of such integration is difficult because the genome of mature human sperm is highly condensed. The ideal means of studying such integration requires direct visualization of human sperm chromosomes. Mature sperm chromosomes do not appear until sperm fertilize ova, specifically, at male pronuclear formation during the first-cleavage division [20], [21]. In this way, human ova are indispensable for direct analysis of sperm chromosomes. However, it is extremely difficult to obtain human ova in sufficient number for meaningful experiments. Such analyses using the human sperm and the human ova also raise ethical issues. Fortunately, great advances have been made in reproductive and developmental biology. In 1978, Rudak et al described a technique that allowed the direct visualization of human sperm chromosomes. This method which involved the in-vitro penetration of zona-free ova of hamsters by capacitated human spermatozoa and the subsequent fixation of the pronuclear chromosomes. This avoided the physical difficulty and ethical issues of obtaining human ova. It has become the only method readily available for direct analysis of human sperm chromosomes.

The above techniques were here used to prepare the sperm chromosome of HIV/AIDS patients. In-vitro penetration of zona-free hamster ova by capacitated patient spermatozoa took place successfully, and sperm chromosomes were prepared. Then the FISH method was used on these chromosomes. The integrated HIV-1 provirus was detected on the chromosomes of spermatozoa from HIV/AIDS patients but not on chromosomes from control. This supports a prior finding that integrated HIV DNA can be detected in purified sperm cells of HIV seropositive subjects through Alu-LTR PCR assay, demonstrating that the HIV-1 provirus can integrate into the chromosomes of germ cells. Several previous studies have also shown that human sperm can spontaneously internalize the viral nucleic acids and then integrate the sequence into the spermatozoa genome [22]–[24]. This to an extent, also supports the current result.

### Transfer of HIV-1 into ova and its replication in early embryo

For the reasons mentioned above, this study on the transfer of HIV-1 into ova and its replication in early embryos could not be carried out in ordinary human embryos. In the current study, we performed the inter-specific fertilization of patient's sperm and zona-free hamster ova. This was expected to have same meaning as two-cell embryo assays performed on IVF human sperm and human ova. One hundred and seventeen 2-cell embryos were successfully collected. This indicated first that the HIV-1 infected spermatozoa had not been eliminated selectively and had the ability to fertilize the ova. The nuclei were prepared from fifty six 2-cell embryos and then FISH analysis revealed the presence of HIV-1 provirus in 1 of 2 nuclei from three 2-cell embryos and in each nucleus from 1 2-cell embryo. The results further confirmed the transfer of the sperm-introduced HIV-1 provirus into 2-cell embryos and proved that the integrated HIV genes can replicate themselves in the early embryonic cells.

In 1994, Baccetti et al. used immunocytochemistry and in situ hybridization at the electron microscopy level, and, using PCR, they observed that HIV-1 can bind to and enter normal sperm [10]. They saw that viral particles, their antigens, and their nucleic acids are present in the cytoplasm of sperm from HIV-1 infected men and that such sperm can transfer HIV-1 like particles to normal human oocytes. However, it is still unclear whether HIV-1 genes can integrate into sperm genome of HIV-1 patients, and whether sperm-introduced HIV-1 genes retain their replication capability in embryos. In the current study, our results not only detected HIV-1 genes in the sperm genome of HIV-1 patients but also demonstrated that the sperm-introduced HIV-1 genes were able to replicate themselves alongside host embryo genome. The two copies were segregated into the two daughter cells during the first cleavage.

### The expression of the HIV-1 gene in sperm and embryos

One previous study suggested that HIV-1 particles in patient sperm could be transferred into early embryos by fertilization [10]. However, it remains unclear whether sperm-introduced HIV-1 genes continue to function after being transferred into embryos by fertilization. In this study, we analyzed 91 human-hamster interspecific 2-cell embryos derived from spermatozoa of HIV/AIDS patients and zona-free oocytes of hamster. IFA was used to detect the expression of HIV-1 P24 in both sperm and embryos. IFA results revealed the presence of HIV-1 p24 in both patient spermatozoa and embryos, indicating expression of the HIV-1 gene in patient spermatozoa and in early embryos derived from patient sperm.

In conclusion, we confirmed the presence of HIV-1 nucleic acids and proteins in spermatozoa and the ability of these HIV-1-infected spermatozoa to fertilize ova, and transfer HIV-1 into early embryos. More importantly, this study provides solid evidence supporting some new views that (1) the HIV-1 provirus can integrate into the genome of germ cells in male patients, including sperm cells, that (2) the integrated HIV-1 provirus in sperm can be transferred into the embryo by fertilization and can replicate itself along with the embryonic genome during the first cleavage division in the early embryo; and that (3) HIV-1 can express proteins after the integrated HIV-1 provirus in the patient sperm had been transferred into early embryos. The results are similar to those of one of our previous studies, in which sperm from healthy donors were transfected with recombinant plasmid pIRES2-EGFP-gag [25]. In the current study the sperm are from HIV-1 patients. These results therefore more truly reflect the nature of these biological events.

Taken together, our study provides several prerequisites for the vertical transmission of HIV-1 by sperm, many of which will have great significance in clinical and basic research.

## Methods

### Ethical approval

This study was carried out in strict accordance with the guidelines for kindly treatment of laboratory animals issued by the Ministry of Science and Technology of China and with administrative regulations of Guangdong Province. For further information please see http://www.labagd.com/codeStandardIndex.html. The protocol was approved by the Institutional Ethical Review Board (IERB) of Shantou University Medical College (Permit number: UMC: 2009-007). All surgeries were performed under sodium pentobarbital anesthesia, and all efforts were made to minimize suffering. Informed consents were provided by all human subjects. The human subject protocol was approved by an IERB, that subjects were fully informed of the research aims. Fertilization of hamster ova with human sperm is a commonly-used methodology, and that all embryos were destroyed at the 2-cell stage without being transferred into any animal [20].

### Subject

Thirty-three male HIV/AIDS patients, who had been confirmed to be HIV-positive by Western blot analysis at the Center for Disease Control and Prevention (CDC, Chongqing, China), were enrolled. Patient ages ranged from 24 to 49, with an average age of 31.8 years. Most of them (96.9%) had acquired the virus through sexual transmission, mainly through homosexual contact (81.8%). At the time of semen donation, 7 patients had received highly active antiretroviral therapy (HAART). Two patients respectively exhibited AIDS symptoms of pneumocystis pneumonia and oral fungal infection, respectively. None was azoospermic, and semen samples contained varying number of motile spermatozoa. Three healthy donors whose semen was of normal quality were enrolled in this study as controls.

### Insemination and post- insemination culture procedures

Semen samples were obtained by masturbation from male HIV/AIDS patients after a recommended 3-day abstinence period. Samples were kept in a CO_2_ incubator (37°C, 50 ml/L CO_2_ in air) for 30 min to allow liquefaction. Motile spermatozoa were selected by the swim-up method and pellets of motile sperm were obtained by centrifugation at 300 g for 5 min. Each sperm pellet was resuspended in BWW medium with 0.3% BSA to obtain a final concentration of 1×10^6^ sperm/ml for fertilization use [25]. The adult 6-to-8-week-old female golden hamsters were maintained under standard laboratory conditions (14 h light: 10 h darkness cycle). Ova were collected from golden hamsters as described previously [26].

Insemination was undertaken with the patients sperm suspensions at a concentration of 10^6^/ml. The oocytes were kept in the sperm suspension for 20–30 min and then transferred and incubated in BWW medium with 0.3% BSA under mineral oil (Sigma) for another 1 h to ensure sperm penetration. Then the ova were washed twice in ovum culture medium (OCM, Flow Laboratories, Germany) containing 15% heat-inactivated fetal bovine serum (as below), and then transferred into a Petri dish with 1.5 ml OCM under mineral oil for post-insemination culture. Ova were separated into two groups. One group was incubated for 24 h until the formation of 2-cell embryos. The other group was incubated for 8 h. Then podophyllotoxin (Sigma) and vinblastine (Sigma) was gently added to the medium to a final concentration of 0.04 µg/ml of both chemicals in order to block karyogamy and first-cleavage spindle formation of the ova. Sixteen hours later, many eggs had reached metaphase and their pronuclei became invisible under the dissecting microscope.

### Preparation of inter-phase nuclei spermatozoa

The inter-phase nuclei sperm was prepared as follows. First, liquefied semen samples from the patients were centrifuged at 600×g for 5 min at room temperature, and the sperm pellets were washed twice with phosphate-buffered saline (PBS). Then the sperm were incubated with PBS containing 2 mM dithiothreitol (DTT) for 45 min to permit decondensation. Next, the sperm were fixed twice in fixative (methanol: acetic acid = 3∶1) for 20 min. After centrifugation, the sperm pellets were resuspended in newly prepared fixative (methanol∶acetic acid = 1∶1), and the sperm suspensions were dropped immediately onto pre-cooled slides. These slides were air-dried and stored at room temperature for later use. Inter-phase nuclei spermatozoa from healthy donors were prepared by the same procedures and served as controls.

### Preparation of sperm chromosomes and inter-phase nuclei of embryo

The human sperm chromosomes and inter-phase nuclei of embryos were prepared using a gradual fixation-air-dry method, as described previously [21]. Briefly, about 5∼10 zygotes or 2-cell embryos were treated with hypotonic solution (0.9% sodium citrate in distilled water containing 3% fetal bovine serum) at 37°C for about 30 min. Then they were transferred into fixative (I) (methanol∶acetic acid: H_2_O = 5∶1∶2) for about 7 min. When the color of the zygotes or embryos changed from brown to white and became subtransparent, these cells and a small amount of fixative(II) (methanol∶acetic acid = 3∶1) were aspirated and released into a circle that was marked on the reverse side of a grease-free slide, and immediately covered by a gentle flow of fixative (II). The slide was immediately placed into a Coplin jar filled with fixativeII and kept there for at least 5 min. Finally, the slide was dipped carefully into fixativeIII (methanol∶acetic acid: H_2_O = 3∶3∶1) for 1 min and dried with warm moist air.

### Fluorescence In situ Hybridization (FISH)

*HIV-1 DNA probes labeling with fluorescein isothiocyanate (FITC)*: HIV-1 gag and pol DNA was PCR-amplified from plasmid ΔNRF of a lentivirus vector. The primers for gag DNA PCR amplification were as follows: ATGGGTGCGAGAGCGTCA (forward) and TGCCCCCCTATCTTTATTGTGA (reverse). The primers for pol DNA amplification were as follows: TCACTCTTTGGCAGCGACC (forward) and CCATGTGTTAATCCTCA TCCTGTCT (reverse). Amplified DNAs were purified then labeled with biotin-14-dATP by nick translation (BioNickTM Labeling System, Invitrogen, Shanghai, China) according to the manufacturer's protocol. Labeled oligonucleotides were purified using the same method mentioned above, and then stored at −20°C for later use.

*In situ hybridization*: FISH was performed to assess the integration of HIV-1 DNA in metaphase chromosomes of spermatozoa from HIV/AIDS patients and in the nuclei of 2-cell embryos derived from HIV/AIDS patient spermatozoa. Spermatozoa of healthy donors and embryos derived from these spermatozoa served as controls. The hybridization processes was performed as described previously [27].

### Indirect immunofluorescence assay (IFA)

2-cell embryos were collected and washed twice with PBS, then fixed in 4% paraformaldehyde in PBS for 15min at room temperature. After fixation, the embryos were washed and permeabilized with a mixture containning 0.5% Triton X-100, 0.5 mM MgCl2, 5 mM EGTA and 50 µM glycine for 15 min, washed again and incubated with blocking solution (0.1% phosphate buffer with 2% fetal calf serum and 10% goat serum) at 4°C for 1 h. Then, the embryos were incubated with primary mouse monoclonal anti-HIV-1p24 antibody(1∶50, as the recommendation of manual; Santa Cruz, USA) at 37°C for 2 h, washed three times in 1×PBS and then incubated with FITC-conjugated rabbit immunoglobulin (diluted in the same way as primary antibody, SANTA CRUZ, USA) at 37°C for 40 min. After being washed three times, the embryos were counterstained with 50 µg/mL PI for 5 min. The embryos were then transferred onto slides and observed by fluorescence microscopy. For comparison, embryos derived from the spermatozoa of healthy donors were similarly analyzed.

IFAs were also performed to detect the expression of HIV-1 p24 in spermatozoa of HIV/AIDS patients and healthy donors (negative control).
